# Methionine biosynthesis and transport are functionally redundant for the growth and virulence of *Salmonella* Typhimurium

**DOI:** 10.1074/jbc.RA118.002592

**Published:** 2018-05-02

**Authors:** Asma Ul Husna, Nancy Wang, Simon A. Cobbold, Hayley J. Newton, Dianna M. Hocking, Jonathan J. Wilksch, Timothy A. Scott, Mark R. Davies, Jay C. Hinton, Jai J. Tree, Trevor Lithgow, Malcolm J. McConville, Richard A. Strugnell

**Affiliations:** From the ‡ Department of Microbiology and Immunology, University of Melbourne at the Peter Doherty Institute for Infection and Immunity, Parkville, Victoria 3000, Australia,; the § Department of Biochemistry and Molecular Biology, University of Melbourne at the Bio21 Institute, Parkville, Victoria 3052, Australia,; the ¶ Institute of Integrative Biology, University of Liverpool, Liverpool L69 7ZB, United Kingdom,; the ‖ School of Biotechnology and Biomolecular Sciences, University of New South Wales, Sydney, New South Wales 2052, Australia, and; the ** Department of Microbiology, Monash University, Clayton, Victoria 3800, Australia

**Keywords:** methionine, S-adenosylmethionine (SAM), biosynthesis, transporter, Salmonella enterica, peptidoglycan, in vivo infection, virulence

## Abstract

Methionine (Met) is an amino acid essential for many important cellular and biosynthetic functions, including the initiation of protein synthesis and *S*-adenosylmethionine–mediated methylation of proteins, RNA, and DNA. The *de novo* biosynthetic pathway of Met is well conserved across prokaryotes but absent from vertebrates, making it a plausible antimicrobial target. Using a systematic approach, we examined the essentiality of *de novo* methionine biosynthesis in *Salmonella enterica* serovar Typhimurium, a bacterial pathogen causing significant gastrointestinal and systemic diseases in humans and agricultural animals. Our data demonstrate that Met biosynthesis is essential for *S.* Typhimurium to grow in synthetic medium and within cultured epithelial cells where Met is depleted in the environment. During systemic infection of mice, the virulence of *S.* Typhimurium was not affected when either *de novo* Met biosynthesis or high-affinity Met transport was disrupted alone, but combined disruption in both led to severe *in vivo* growth attenuation, demonstrating a functional redundancy between *de novo* biosynthesis and acquisition as a mechanism of sourcing Met to support growth and virulence for *S.* Typhimurium during infection. In addition, our LC-MS analysis revealed global changes in the metabolome of *S.* Typhimurium mutants lacking Met biosynthesis and also uncovered unexpected interactions between Met and peptidoglycan biosynthesis. Together, this study highlights the complexity of the interactions between a single amino acid, Met, and other bacterial processes leading to virulence in the host and indicates that disrupting the *de novo* biosynthetic pathway alone is likely to be ineffective as an antimicrobial therapy against *S.* Typhimurium.

## Introduction

Methionine (Met) is a sulfur-containing proteinogenic amino acid that is required for the initiation of protein synthesis (1). *S*-adenosylmethionine (SAM),^7^ a downstream derivative of Met, acts as a major methyl donor in the cell and methylates a variety of macromolecules, such as DNA, RNA, protein, and lipids (2). A *de novo* pathway for Met biosynthesis is present in the vast majority of prokaryotes, albeit with variations in the enzymes that drive the biosynthetic cascade (3, 4). In contrast, the full *de novo* Met biosynthesis is absent from vertebrates, which must obtain this amino acid through external sources, such as diet and gut flora (5, 6). In recent years, it has been increasingly recognized that central metabolism represents a promising yet underexploited area for the development of antimicrobial drugs (7). The apparent essentiality in microbes and absence in mammals makes the Met biosynthetic pathway an especially attractive target for antimicrobial therapy.

*Salmonella enterica* is a Gram-negative, facultative intracellular bacterium that causes gastrointestinal and systemic diseases in animals and humans. There are ∼2500 serovars in this species, which include the human-adapted enteric fever pathogens *S. enterica* var. Typhi and Paratyphi, alongside non-typhoidal *Salmonella* (NTS) serovars that cause debilitating gastroenteritis, including *S. enterica* var. Typhimurium (8, 9). For all *Salmonella* serovars, the capacity to grow in the host is central to bacterial virulence (10). In mammalian hosts, *S. enterica* grows in the blood and reticuloendothelial system largely within a defined, membrane-bound endocytic compartment called the *S**almonella-*containing vacuole (SCV) (11, 12). The extent to which *S. enterica* salvages essential nutrients from the lumen of the SCV and the nutrient composition of this vacuole remains poorly defined, and the micronutrient environment of the SCV has not been fully resolved. *Ex vivo*, *S. enterica* is capable of growth in minimal medium containing glucose as a carbon source and key ions, and the metabolic potential of this species has been recognized and mapped (13). It is less clear, however, how this considerable capability is exploited for maximal growth *in vivo*. The essentiality of the pathways can only be determined by systematic analysis *in vitro* and in animal models, and fine mapping is required to determine whether essential pathways contain novel targets for antibiotic development.

**Figure 1.**
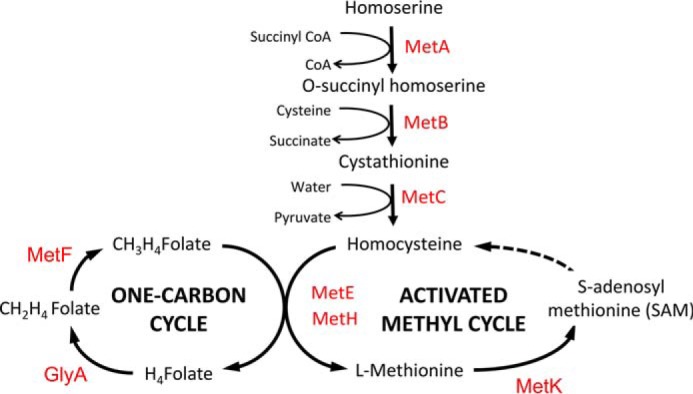
**Schematic diagram of the biosynthetic pathway of Met in *S.* Typhimurium.** The first committed step of the pathway is catalyzed by homoserine transsuccinylase (MetA), which succinylates homoserine to form *O*-succinyl-l-homoserine, which then undergoes a condensation reaction with cysteine to form l-cystathionine, catalyzed by cystathionine γ-synthase (MetB). Cystathionine β-lyase (MetC) catalyzes the conversion of l-cystathionine to l-homocysteine, pyruvate, and ammonia. The final biosynthetic step is catalyzed by distinct Met synthases, MetH and MetE, which are vitamin B_12_-dependent and -independent synthases, respectively (31, 32, 64). In this final step, l-homocysteine is condensed with 5,10-methyltetrahydrofolate (*CH_3_H_4_Folate*), which donates the methyl group. 5,10-Methyltetrahydrofolate is provided from the one-carbon cycle through a methylenetetrahydrofolate reductase (MetF)-mediated reaction, producing tetrahydrofolate (*H_4_Folate*) and Met (65). In *S. enterica*, Met is recycled through an activated methyl cycle. The primary methyl donor, SAM, is formed by the activation of Met through an ATP-dependent condensation reaction catalyzed by the SAM synthase (MetK)(2, 66, 67). Catalytic enzymes are shown in *red*; *arrows* indicate the direction of catalytic reactions.

The *de novo* biosynthetic pathway for Met has been previously reported in *S. enterica* (Fig. 1). This pathway is regulated by the transcription factors MetJ and MetR (14). With its co-repressor SAM, MetJ represses the transcription of all *met* genes except *metH* (15). In contrast, MetR is an autoregulated transcriptional activator that controls the expression of *metA*, *metF*, *metE*, and *metH* (16). In addition to *de novo* biosynthesis, *S. enterica* is able to acquire Met from extracellular sources through a high-affinity transporter that is encoded by the *metD* locus, encoding an ATP-binding cassette (ABC) transporter composed of three subunits: the ATPase (MetN), a transmembrane permease (MetI), and a periplasmic Met-binding protein (MetQ). Biochemical analysis has shown that MetNIQ transports both the d- and l-enantiomers of Met (17, 18). In the absence of the high-affinity MetNIQ transporter system, genetic analysis has suggested that *S. enterica* is able to transport Met at much lower affinity, through a putative and cryptic low-affinity Met transporter system termed “MetP” (19–21). In contrast to MetNIQ, MetP transports l-Met but not d-Met, and the coding gene(s) for MetP remains unidentified to date.

*S. enterica* may encounter markedly different nutrient levels during local (*e.g.* the gut) and systemic (*e.g.* the spleen and liver) infections, which, in turn, may vary the bacterium's dependence on *de novo* biosynthesis and nutrient import pathways for growth in different tissue niches. The enzymes required for Met biosynthesis and the Met transporter have been implicated in the virulence of *S. enterica* and other bacteria (22–28). However, these pathways have not been systematically investigated for their role in the virulence of *S. enterica*. The aim of this study was to investigate the essentiality of *de novo* biosynthesis and transport of Met in the growth and virulence of *S.* Typhimurium *in vitro* and *in vivo*.

## Results

### In vitro validation of the Met biosynthetic pathway in S. Typhimurium and growth of defined Met auxotrophs in mice

Previous transcriptional analyses of *S.* Typhimurium grown in medium and in tissue culture cells have demonstrated that the genes involved in the *de novo* Met synthesis pathway (*metA*, *metB*, *metC*, *metE*, *metF*, and *metH*) are expressed under a variety of growth conditions (Fig. S1; deduced from Ref. 29). Whereas the expression level of the *de novo* pathway is relatively low in rich medium, much stronger expression was observed under growth conditions where the *Salmonella* pathogenicity island-2 (SPI-2) genes were expressed or when the bacteria were inside macrophages (30).

**Figure 2.**
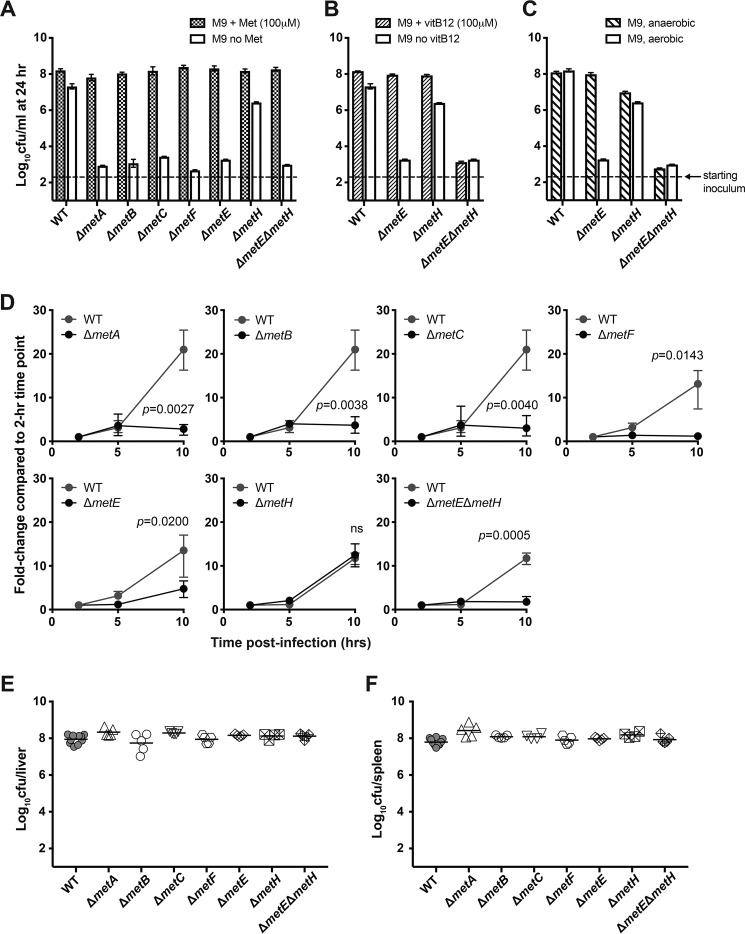
**The *de novo* biosynthetic mutants demonstrate Met-dependent growth in M9 minimal medium and in HeLa cells but remain fully virulent in mice.** *S.* Typhimurium WT SL1344 and mutant strains were grown shaking at 37 °C in M9 minimal medium with or without Met (*A*), vitamin B_12_ (*B*), or oxygen (*C*), and the number of viable bacteria (cfu) was determined after 24 h. *Bars*, mean cfu; *error bars*, data range. *Dotted lines* represent the bacterial concentration at the time of inoculation. Data are pooled from two independent experiments. *D*, the *de novo* Met biosynthetic mutants become defective for intracellular replication in HeLa cells when Met is absent in the DMEM. HeLa cells were grown to a monolayer and infected with *S.* Typhimurium WT or mutant strains at an MOI of 5–10, in Met-free DMEM. The intracellular bacterial load at 2 h post-infection is expressed as “1” and used as the reference point to calculate -fold change of intracellular bacterial number at subsequent time points. Data are pooled from three independent experiments. *Bars*, mean cfu; *error bars*, data range. Unpaired *t* tests were used to compare the intracellular load of WT and mutant strains at 10 h post-infection, and *p* values shown were corrected for multiple comparisons using the Bonferroni–Dunn method; *p* values greater than 0.05 were deemed not significant (*ns*). For assessing virulence *in vivo*, age- and sex-matched C57BL/6 mice were intravenously infected with 200 cfu of the indicated strains of *S.* Typhimurium, and the bacterial load in the liver (*E*) and spleen (*F*) was determined at day 5 post-infection. *Symbols* represent data from individual animals, and *horizontal lines* represent the geometric mean of each group. Data are pooled from two independent experiments. One-way ANOVA with Bonferroni post-tests was used for statistical analyses comparing each pair of data groups, and none of the comparisons yielded a *p* value < 0.05.

Defined mutants in the Met biosynthetic pathway (Δ*metA*, Δ*metB*, Δ*metC*, Δ*metE,* Δ*metF*, Δ*metH*, and Δ*metE*Δ*metH*) were generated in *S.* Typhimurium SL1344, sequenced, and tested for their ability to grow in M9 minimal medium, with or without added l-Met, and in nutrient-rich Luria–Bertani (LB) medium. All mutants grew in LB medium at a rate comparable with WT SL1344 (data not shown). In M9 minimal medium supplemented with Met, Met mutants grew as efficiently as SL1344 over a 24-h period (Fig. 2*A*), suggesting that the absence of the *de novo* biosynthetic pathway was compensated by the transport of exogenously available Met. As expected, the Δ*metA*, Δ*metB*, Δ*metC*, Δ*metE*, and Δ*metF* mutants exhibited Met auxotrophy in M9 medium, whereas Δ*metH* showed substantial growth over the 24-h period (Fig. 2*A*), confirming that MetH is not essential for *de novo* biosynthesis of Met under aerobic conditions (31, 32). The growth of the Δ*metH* mutant in M9 medium was apparently supported by a functional Met synthase (*i.e.* MetE), as Δ*metE*Δ*metH* failed to grow in M9 medium in the absence of exogenous Met (Fig. 2*A*). It has been suggested that the functionality of MetH in *E. coli* is vitamin B_12_–dependent (33). To test whether there is an equivalent dependence in *S.* Typhimurium, Δ*metE*, Δ*metH*, and Δ*metE*Δ*metH* mutants were grown in M9 medium with or without vitamin B_12_. All three mutants grew in the presence of vitamin B_12_ (Fig. 2*B*). The Δ*metE* mutant, which depended on MetH to synthesize Met, grew only when vitamin B_12_ was added (Fig. 2*B*), confirming that MetH activity in *S.* Typhimurium is vitamin B_12_–dependent. As *S*. Typhimurium synthesizes vitamin B_12_ only under anaerobic conditions (31), growth of the Δ*metE* mutant in M9 medium should have been restored when oxygen was depleted. Indeed, Δ*metE*, Δ*metH*, and Δ*metE*Δ*metH* both grew in M9 medium under anaerobic conditions and phenocopied their growth in M9 medium with vitamin B_12_ under aerobic conditions (Fig. 2*C*). These observations are consistent with an understanding that *S.* Typhimurium synthesizes vitamin B_12_ under anaerobic conditions and support a level of functional redundancy between MetE and MetH during *de novo* biosynthesis of Met in *S.* Typhimurium.

To assess the requirement for *de novo* Met synthesis for intracellular bacterial growth, HeLa cells were infected with *S.* Typhimurium WT and mutants, and the intracellular bacterial load was assessed after 2, 5, and 10 h. When HeLa cells were cultivated in DMEM growth medium lacking Met, the intracellular growth of Δ*metA*, Δ*metB*, Δ*metC*, and Δ*metF* was significantly attenuated compared with the WT control (Fig. 2*D*). The Δ*metE* mutant only showed obvious growth after 5 h. In contrast, the Δ*metH* mutant showed intracellular growth comparable with SL1344, suggesting that a functional MetE is sufficient for supporting intracellular replication in the SCV (Fig. 2*D*). Importantly, when HeLa cells were cultivated in DMEM containing physiological levels of l-Met, none of the mutants showed any significant defect in intracellular growth (Fig. S2), demonstrating that exogenous Met can rescue Met auxotrophy, presumably reflecting direct or indirect transport of Met from the culture medium to the lumen of the bacteria-occupied SCV.

Previous reports have proposed that Met biosynthesis might be required for virulence of *S.* Typhimurium in mice or chickens (24, 34). To test this requirement in mice, the virulence of individual *de novo* biosynthetic mutants was studied in C57BL/6 mice, in which WT SL1344 is fully virulent and invariably results in a systemic, lethal infection (35, 36). Groups of mice were infected with 200 cfu of the different Met auxotrophic mutants intravenously, and, at day 5 post-infection, mice were culled, and the bacterial load in the liver (Fig. 2*E*) and spleen (Fig. 2*F*) was determined by viable count. The number of bacteria recovered from mice infected with the mutants was comparable with those from mice infected with SL1344. Hence, none of the mutants showed virulence defects in mice following intravenous infection. Another group of mice were infected orally with Δ*metB*, Δ*metE,* Δ*metH*, or Δ*metE*Δ*metH*. The bacterial load in the spleen and liver of mice infected with the mutant strains was comparable with those from mice infected with the WT *S.* Typhimurium (Fig. S3). These data showed that *de novo* Met biosynthesis is not essential for the growth of *S*. Typhimurium in C57BL/6 mice, regardless of the route of infection.

### Met auxotrophs deficient in the high-affinity transporter (MetNIQ) are attenuated in mice

**Figure 3.**
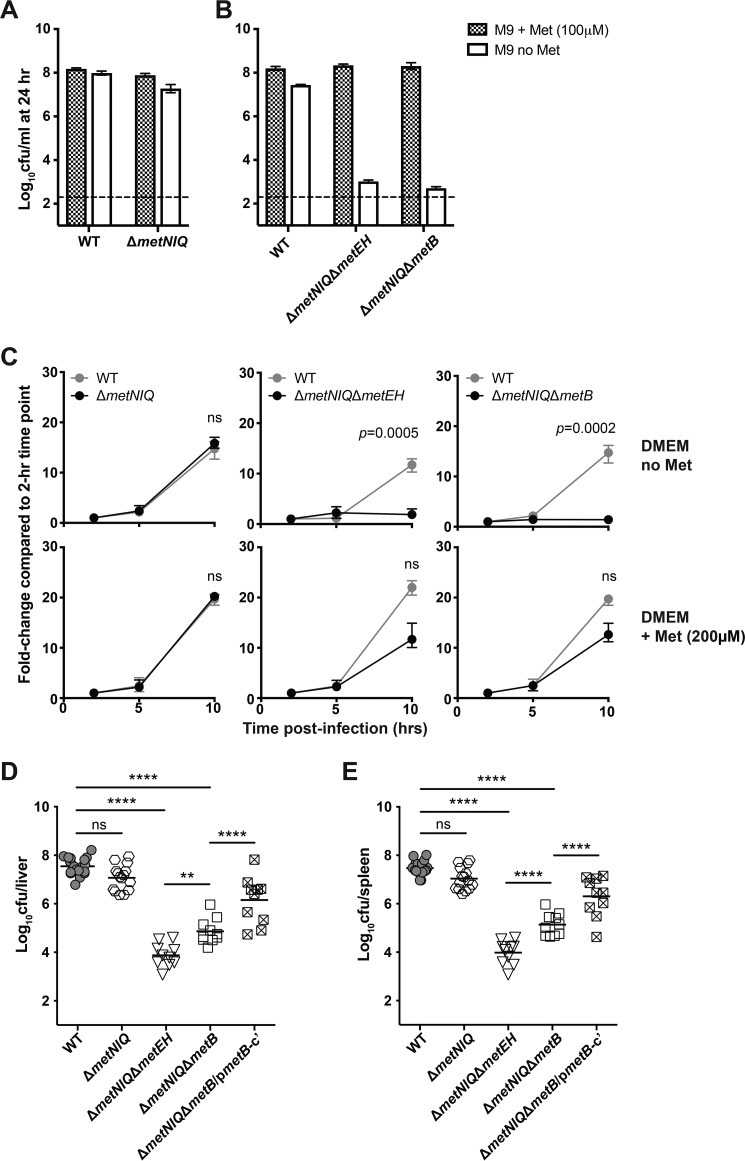
***S.* Typhimurium mutants deficient in the *de novo* biosynthesis and high-affinity transport of Met are highly attenuated in mice.** *A* and *B*, *S.* Typhimurium strains were grown shaking at 37 °C in M9 minimal medium with or without Met, and the number of viable bacteria (cfu) was determined after 24 h. *Bars*, mean cfu; *error bars*, data range. *Dotted lines*, bacterial concentration at the time of inoculation. Data are pooled from two independent experiments. *C*, HeLa cells were grown to a monolayer and infected with *S.* Typhimurium WT or mutant strains at an MOI of 5–10, in DMEM with or without Met. The intracellular bacterial load at 2 h post-infection is expressed as “1” and used as the reference point to calculate -fold change of intracellular bacterial number at subsequent time points. *Bars*, mean cfu; *error bars*, data range. Data are pooled from three independent experiments. Unpaired *t* tests were used to compare the intracellular load of WT and mutant strains at 10 h post-infection, and *p* values shown were corrected for multiple comparisons using the Bonferroni–Dunn method; *p* values > 0.05 were deemed not significant (*ns*). For assessing virulence *in vivo*, age- and sex-matched C57BL/6 mice were intravenously infected with 200 cfu of the indicated strains of *S.* Typhimurium, and the bacterial load in the liver (*D*) and spleen (*E*) was determined at day 5 post-infection. Δ*metNIQ*Δ*metB*/p*metB*-c′ denotes the complemented strain Δ*metNIQ*Δ*metB* pACYC184*metB. Symbols* represent data from individual animals, and *horizontal lines* represent the geometric mean of each group. Data are pooled from three independent experiments. One-way ANOVA with Bonferroni post-tests was used for statistical analyses. ****, *p* < 0.0001; *ns*, *p* > 0.05.

Our observation that *S.* Typhimurium mutants were able to grow intracellularly and *in vivo* without a functional *de novo* Met biosynthetic pathway suggested that Met acquisition via the transporters may play an important role in bacterial virulence. To test this hypothesis, a high-affinity transporter mutant of *S*. Typhimurium, Δ*metNIQ*, was constructed. As expected, the Δ*metNIQ* mutant grew in M9 minimal medium in the absence of Met supplementation (Fig. 3*A*), suggesting that the high-affinity Met transporter is not essential for growth in M9 medium provided that *de novo* synthesis is intact.

To determine whether dual mutations in *de novo* biosynthesis and the high-affinity transporter of Met affected the growth of *S.* Typhimurium *in vitro* and *in vivo*, Δ*metNIQ*Δ*metEH* and Δ*metNIQ*Δ*metB* mutants were generated. As expected, the Δ*metNIQ*Δ*metEH* and Δ*metNIQ*Δ*metB* mutants were unable to grow in M9 minimal medium without added Met (Fig. 3*B*); this Met auxotrophy confirms that a combined deficiency in both biosynthesis and high-affinity transport is inhibitive for growth. Interestingly, the addition of l-Met into M9 medium restored the growth of all of these mutant strains, consistent with the presence and activity of the putative and functional low-affinity transporter MetP (19, 20). Hence, these data support the theory that MetP activity enables sufficient Met uptake to facilitate efficient growth *in vitro*.

To determine whether the observed *in vitro* growth attenuation was reflected by reduced growth of *S.* Typhimurium inside mammalian cells, Δ*metNIQ*, Δ*metNIQ*Δ*metB*, and Δ*metNIQ*Δ*metEH* mutants were used to infect HeLa cells in Met-free DMEM. Whereas Δ*metNIQ* mutant was capable of intracellular growth to a level similar to SL1344 over a 10-h period, Δ*metNIQ*Δ*metB* and Δ*metNIQ*Δ*metEH* mutants were unable to grow (Fig. 3*C*, *top row*). The presence of l-Met in DMEM fully restored the growth of the mutant strains in HeLa cells (Fig. 3*C*, *bottom row*), suggesting that the activity of the cryptic transporter MetP was sufficient to support intracellular growth in HeLa cells.

To determine whether dual mutations in *de novo* biosynthesis and the high-affinity transporter of Met reduced *S.* Typhimurium virulence *in vivo*, C57BL/6 mice were intravenously infected with Δ*metNIQ*, Δ*metNIQ*Δ*metB*, and Δ*metNIQ*Δ*metEH* mutant strains or WT. At day 5 post-infection, mice infected with Δ*metNIQ* had a similarly high bacterial load in the liver (Fig. 3*D*) and spleen (Fig. 3*E*), comparable with that of SL1344-infected mice, indicating that loss of the high-affinity Met transporter alone does not reduce *S.* Typhimurium virulence *in vivo*. In contrast, infection with Δ*metNIQ*Δ*metB* and Δ*metNIQ*Δ*metEH* mutants resulted in a significantly reduced bacterial load in the liver (Fig. 3*D*) and spleen (Fig. 3*E*), and this attenuation was reversed when the Δ*metNIQ*Δ*metB* mutant was complemented by *metB* (Fig. 3, *D* and *E*). Similar results were obtained when the Δ*metNIQ*Δ*metB* and Δ*metNIQ*Δ*metEH* mutants were used to infect mice via the oral route (Fig. S4), confirming that Δ*metNIQ*Δ*metB* and Δ*metNIQ*Δ*metEH* are attenuated in mice regardless of the route of infection. Taken together, these results demonstrate that restriction of Met availability has a strong impact on the virulence of *S.* Typhimurium *in vivo*, and the presence of the putative cryptic transporter (MetP) alone is insufficient to sustain maximal bacterial growth in the permissive murine host.

### Metabolite profiling in S. Typhimurium Met biosynthetic and high-affinity transporter mutants

**Figure 4.**
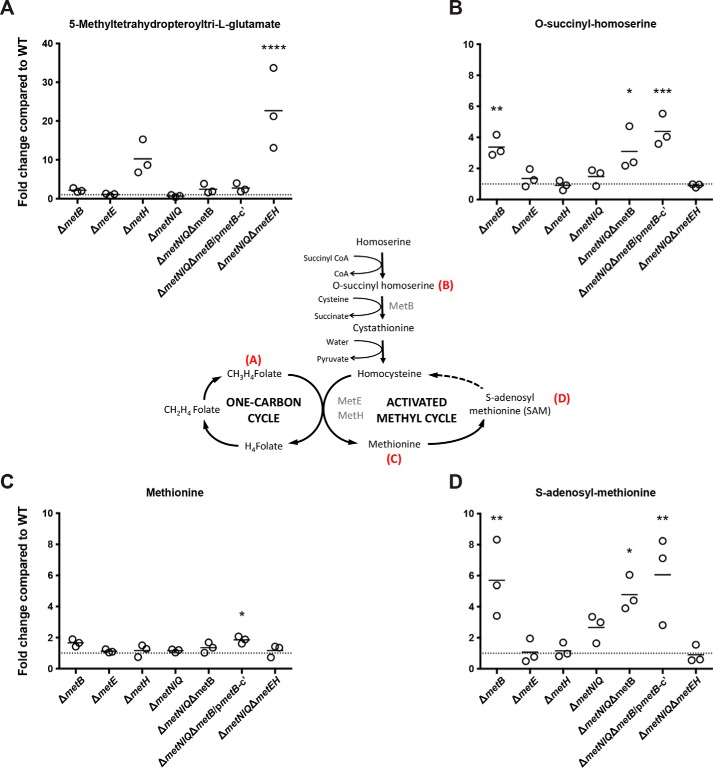
**Metabolite perturbations following genetic disruption to Met biosynthesis, one-carbon cycle, and activated methyl cycle.** Total metabolite pools in each strain were detected and quantitated using liquid chromatography-quadrupole TOF. Key intermediates of the Met biosynthetic pathway, activated methyl cycle, and one-carbon cycle were identified and quantified for the indicated *S.* Typhimurium strains and compared with the WT level; -fold changes are shown for 5-methyltetrahydropteroyltri-l-glutamate, a member of the polyglutamate forms of 5-methyltetrahydrofolate (*A*), *O*-succinylhomoserine (*B*), Met (*C*), and SAM (*D*). Δ*metNIQ*Δ*metB*/p*metB*-c′ denotes the complemented strain Δ*metNIQ*Δ*metB* pACYC184*metB.* Individual data points represent biological replicates, and the mean for each group is shown. One-way ANOVAs with Bonferroni post-tests were used to compare the metabolite level in each mutant with WT; *p* values < 0.05 are shown: *, *p* < 0.05; **, *p* < 0.01; ***, *p* < 0.001; ****, *p* < 0.0001.

To further define the impact of Met pathway gene disruptions on bacterial metabolism, *S*. Typhimurium WT and mutants lacking either or both of the *de novo* biosynthesis pathway and the high-affinity Met transporter were grown in LB medium, and polar metabolites were extracted and analyzed by LC-MS. The intracellular accumulation of 5-methyl-tetrahydropteroyltri-l-glutamate, a member of the polyglutamate forms of 5-methyltetrahydrofolate (37), was significantly increased in Δ*metH* (∼10-fold) and further increased in Δ*metNIQ*Δ*metEH* (∼25-fold), but not in Δ*metE*, compared with SL1344 (Fig. 4). This result suggests that both MetE and MetH are functionally active, as *S.* Typhimurium grew in LB broth, but MetH is either more abundant or more efficient in the formation of 5-methyltetrahydrofolate. On the other hand, the Δ*metB* mutant exhibited a significantly elevated pool of *O*-succinylhomoserine compared with the WT, consistent with a defect in the MetB-catalyzed conversion of *O*-succinylhomoserine to cystathionine (Fig. 4).

**Figure 5.**
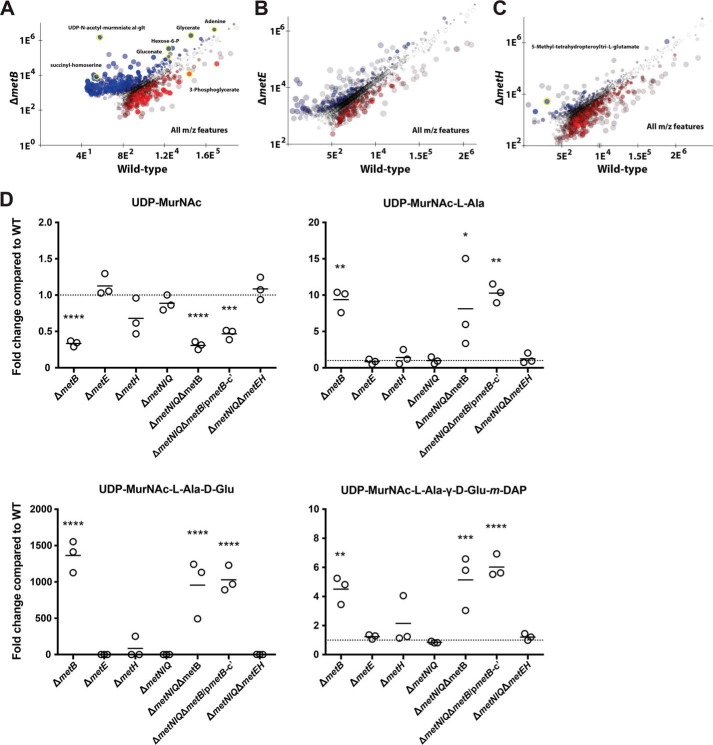
**Metabolite perturbations following genetic disruption to *metB*, *metE*, and *metH*.** Scatter plots depict WT compared with Δ*metB* (*A*), Δ*metE* (*B*), and Δ*metH* (*C*). Each *dot* represents a single extracted *m*/*z* feature with each axis depicting the arbitrary ion count. *m*/*z* features that are no different between conditions plot along the *diagonal*. The *color intensity* indicates the confidence of the difference (*greater intensity* equates to lower variance across biological replicates). *Yellow highlight*, a subset of confirmed metabolites with a statistically significant difference in a pairwise comparison (*p* < 0.05 with Benjamini correction). *D*, the levels of four metabolites associated with peptidoglycan synthesis were significantly altered in Δ*metB* and Δ*metNIQ*Δ*metB* mutants. *UDP-MurNAc*, UDP-*N*-acetylmuraminate; *UDP-MurNAc-*l*-Ala*, UDP-*N*-acetylmuramoyl-l-alanine; *UDP-MurNAc-*l*-Ala-*d*-Glu*, UDP-*N*-acetylmuramoyl-l-alanyl-d-glutamate; *UDP-MurNAc-*l*-Ala-*γ-d*-Glu-m-DAP*, UDP-*N*-acetylmuramoyl-l-alanyl-d-glutamyl-*meso-*diaminopimelate. Δ*metNIQ*Δ*metB*/p*metB-c*′, the complemented strain Δ*metNIQ*Δ*metB* pACYC184*metB.* Individual data points represent biological replicates, and the mean for each group is shown. One-way ANOVAs with Bonferroni post-tests were used to compare the metabolite level in each mutant with WT; *p* values <0.05 are shown: *, *p* < 0.05; **, *p* < 0.01; ***, *p* < 0.001; ****, *p* < 0.0001.

An untargeted mass/charge (*m*/*z*) feature analysis of the mutant lines led to the identification of a number of unexpected metabolite changes. Pairwise comparisons were performed to identify differences that were statistically significant (*p* < 0.05) and to filter interesting *m*/*z* features. Deletion of *metB* led to significant increases in the intracellular levels of SAM (5.5-fold increase compared with WT), and a number of other metabolites, including glycerate, adenine, hexose 6-phosphate, 3-phosphoglycerate, and gluconate, were also increased compared with the WT (Fig. 5*A* and Table 1). By comparison, Δ*metE* did not show any significantly different *m*/*z* features (Fig. 5*B*), whereas only the *m*/*z* feature corresponding to 5-methyl-tetrahydropteroyltri-l-glutamate, a representative form of 5-methyltetrahydrofolate, was significantly different in the Δ*metH* mutant (Fig. 5*C*).

**Table 1:** List of metabolites with significant changes in Δ*metB* mutant compared with WT This table presents results from a one-way ANOVA performed with Tukey's honestly significant difference tests (*p* < 0.01). FDR, false discovery rate.

XCMS ID	Metabolite name	*m*/*z*	-Fold change (Δ*metB* to WT)	*p* value	FDR
M900T1200	4-Hydroxyphenylacetyl-CoA	900.15	3247.21	4.17E − 07	4.01E − 05
M878T1201	UDP-*N*-acetylmuramoyl-l-alanyl-d-glutamate	878.17	1646.67	7.63E − 08	1.45E − 05
M880T1200	3-Hydroxyhexanoyl CoA	880.18	220.30	6.08E − 07	5.15E − 05
M749T1087	UDP-*N*-acetylmuramoyl-l-alanine	749.13	12.24	7.22E − 06	2.49E − 04
M163T480	Rhamnose	163.06	10.30	3.99E − 08	1.33E − 05
M275T1210	6-Phosphogluconate	275.02	8.93	1.01E − 04	1.74E − 03
M105T825	d-Glycerate	105.02	8.15	8.18E − 06	2.67E − 04
M259T1165	Hexose-phosphate	259.02	7.36	7.32E − 04	8.43E − 03
M266T579	Adenosine	266.09	6.13	1.44E − 04	2.33E − 03
M195T999	Gluconate	195.05	6.13	7.04E − 07	5.68E − 05
M1050T1225	UDP-*N*-acetylmuramoyl-l-alanyl-d-γ-glutamyl-meso-2,6- diaminopimelate	1050.26	5.63	1.75E − 06	9.97E − 05
M189T1246	Diaminoheptandioate	189.09	4.73	6.05E − 07	5.15E − 05
M620T1224	UDP-*N*-acetyl-2-amino-2-deoxy-d-glucuronate	620.05	4.67	9.09E − 09	7.57E − 06
M134T607	Adenine	134.05	4.44	6.48E − 06	2.31E − 04
M686T707	Dephospho-CoA	686.14	4.22	1.36E − 05	3.75E − 04
M218T1006	*O*-Succinyl-l-homoserine	218.07	3.75	5.85E − 05	1.14E − 03
M145T567	2-Dehydropantoate	145.05	3.72	2.60E − 05	6.15E − 04
M130T567	Hydoxyproline	130.05	3.19	2.46E − 04	3.57E − 03
M273T1080	Succinyl-arginine	273.12	2.55	1.28E − 04	2.11E − 03
M678T1139	UDP-*N*-acetylmuraminate	678.09	2.45	1.41E − 07	2.10E − 05
M227T987	4-Phosphopantoate	227.03	2.11	3.55E − 04	4.74E − 03
M116T860	Valine	116.07	2.03	1.21E − 04	2.02E − 03

Interestingly, several *m*/*z* features corresponding to peptidoglycan biosynthetic intermediates were significantly different for the Δ*metB* mutant, including UDP-*N*-acetylmuraminate-alanine-glutamate (Fig. 5*D*). This, in turn, suggested an association between Met biosynthesis and peptidoglycan biosynthesis. Disruption of *metB* led to a decrease in intracellular concentrations of UDP-*N*-acetylmuraminate (∼0.4-fold) and a large increase of UDP-*N*-acetylmuramoyl-l-alanine (12-fold), UDP-*N*-acetylmuramoyl-l-alanyl-d-glutamate (1400-fold), and UDP-*N*-acetylmuramoyl-l-alanyl-d-γ-glutamyl-*meso*-2,6-diaminopimelate (4-fold) (Fig. 5*D*). A decrease in UDP-*N*-acetylmuraminate and an increase in UDP-*N*-acetylmuramoyl-l-alanine (9-fold), UDP-*N*-acetylmuramoyl-l-alanyl-d-glutamate (1000-fold), and UDP-*N*-acetylmuramoyl-l-alanyl-d-γ-glutamyl-*meso*-2,6-diaminopimelate (5-fold) was also observed in the double mutant Δ*metNIQ*Δ*metB* (Fig. 5*D*).

*Ex vivo*, the Δ*metB* mutant had a reduced growth rate compared with the WT in bile salts. To study the impact of the altered levels of peptidoglycan-related metabolites, the Δ*metB* mutant and WT were tested in LB broth in the presence of EDTA, SDS, lysozyme, and fasted state–simulated intestinal fluid, but no significant differences in growth were found (Table S1). A small increase (∼2 mm on 15 mm) in β-lactam antibiotic sensitivity, using a validated disc sensitivity method, was observed for the Δ*metB* mutant (data not shown). In addition, when the Δ*metB* and WT were tested in MacConkey broth supplemented with 0.6% bile salt, a small but consistent delay in the growth of Δ*metB* was observed (Fig. S5).

## Discussion

There is increased interest in identifying metabolic pathways in bacterial pathogens, which are essential and distinct from those in their mammalian and human hosts, as potential antibiotic targets (38–42). Previous studies suggested that perhaps 400 *S. enterica* enzymes are dispensable and that essential pathways are often protected against random mutation by redundancy, reflecting the selective pressure placed on metabolism as a key virulence trait (13). Met biosynthesis and transport is an important part of the interconnected and interdependent amino acid metabolism (43). In this study, the essentiality of Met biosynthesis and transport in the mammalian virulence of *S.* Typhimurium was investigated.

We showed that *de novo* Met biosynthesis is not essential when the bacteria are able to obtain Met from their environment. The growth rate of the bacteria with or without *de novo* Met biosynthesis appeared similar in Met-rich media (*e.g.* LB broth), suggesting that transport systems can compensate for the loss of *de novo* biosynthesis. Met biosynthetic mutants require the presence of Met in the culture medium in order to grow inside of HeLa cells (Fig. 2*D* and Fig. S2), suggesting that extracellular Met becomes available to bacteria in the SCV within a few hours of infection. It is likely that Met is relatively abundant in the SCV during *in vivo* infection, as all of the Met biosynthetic mutants were as virulent as WT *S.* Typhimurium in mice (Fig. 2, *E* and *F*), presumably because the Met acquisition systems have access to a sufficient source of Met for supporting bacterial growth in the infected animal.

It is only when *de novo* biosynthesis and high-affinity transport of Met are both disrupted that we observed severe growth attenuation in HeLa cells (Fig. 3*C*) and, importantly, in infected mice (Fig. 3, *D* and *E*). This result strongly suggests functional redundancy between transport and biosynthesis as a source of Met for *S.* Typhimurium inside of the host. Whether Met is acquired (*e.g.* Δ*metB*) or synthesized (*e.g.* Δ*metNIQ* and Δ*metNIQ*Δ*metB* complemented with *metB*), the growth *in vivo* appears to be comparable, suggesting that either *de novo* biosynthesis or the high-affinity transport system of Met alone can provide Met in excess to what is required for *S.* Typhimurium to grow at maximal capacity in the host. Hence, at least in the case of *S.* Typhimurium, it is probably quite difficult to inhibit growth by targeting the Met biosynthetic pathway. The observation that Δ*metNIQ*Δ*metB* and Δ*metNIQ*Δ*metEH* were still able to grow in mice is suggestive of a Met acquisition system independent of MetNIQ, which is consistent with the presence of a low-affinity transporter MetP (19, 20). Hence, our study also provides additional support for the presence of a putative, low-affinity transporter, MetP, which has yet to be identified.

Our description of this apparent redundancy between *de novo* synthesis and high-affinity transport of Met conflicted with previous studies that showed Met auxotrophs are defective for intracellular survival in macrophages and epithelial cells (22, 23) and have reduced virulence in mice (24, 28) and in 1-day-old chickens (34). This may be at least in part due to differences in the study design. In our study, C57BL/6 mice were infected with a single strain, whereas previous studies typically used competition assays (*i.e.* infecting the same host with WT and mutant strains), which may put more pressure on the mutant to grow in the competitive environment. It is not uncommon to have divergent results from single infections and competitive infections (44, 45). Another study has shown that the *S*. Typhimurium lacking the high-affinity transporter MetNIQ is attenuated in C3H/HeN mice (25). C3H/HeN mice carry the resistant allele of *Nramp1*, which controls Fe^3+^ availability in the phagosome and is a major determinant of murine susceptibility to *S.* Typhimurium; consequently, C3H/HeN mice are much more resistant to *S.* Typhimurium infection than the *Nramp1*-deficient C57BL/6 mice (46, 47). It is possible that the differences in attenuation observed in earlier studies, but not ours, relate to differences in genetic susceptibility of the mice. Finally, earlier animal studies did not complement the bacterial Met mutation nor sequence the mutant strains, and it is possible that secondary mutations, not mutations in Met biosynthesis *per se*, were responsible for the observed attenuation in mice.

To generate novel insights about the Met biosynthesis pathway in *S*. Typhimurium, the changes in bacterial concentrations of key metabolites were examined by LC-MS (Figs. 4 and 5). Mass spectrometry revealed substrate accumulation in Met biosynthetic mutants Δ*metB*, Δ*metE*, Δ*metH*, and the high-affinity transporter mutant Δ*metNIQ* and mutants deficient in both (Δ*metNIQ*Δ*metB* and Δ*metNIQ*Δ*metEH*). This analysis revalidated the pathway model for Met biosynthesis shown in Fig. 1, which has been proposed since the 1980s but to the best of our knowledge, has not been systematically studied in *S. enterica* until now.

The observed accumulation of substrates provides interesting insights into the kinetics of enzyme activities. The deletion of both Met synthases (*i.e.* Δ*metNIQ*Δ*metEH*) leads to a profound difference in 5-methyltetrahydrofolate concentration, a metabolite in the one-carbon cycle (Fig. 4). In the Δ*metH* mutant, there is a 10-fold higher intracellular concentration of the substrate compared with Δ*metE*, indicating that MetE is much less efficient than MetH, supporting previous findings (48). Presumably, perturbation of the one-carbon cycle led to an accumulation of homocysteine, which is toxic for bacterial cells (49, 50). This is probably why we consistently observed that Δ*metNIQ*Δ*metEH* grew slower *in vivo* compared with Δ*metNIQ*Δ*metB* (Fig. 3 and Fig. S4).

The disruption of the *metB* gene led to several unexpected observations. First, the intracellular pool of SAM was increased in Δ*metB* compared with WT (Fig. 4), indicating a defect in SAM catabolism following perturbation of the activated methyl cycle in this mutant. This observation argues that in the Δ*metB* mutant, homocysteine, which is converted into SAM, cannot be derived through *de novo* biosynthesis in Δ*metB* and hence is not fed into the activated methyl cycle. Further comparisons of the LC-MS/MS data between Δ*metB* and SL1344 revealed differences in many metabolites from disparate pathways; how disruption to a single enzyme in Met biosynthesis caused perturbations in other pathways was unclear, but the data demonstrate the complexity of modeling intracellular metabolite fluxes in bacteria. Interestingly, this analysis revealed that the concentrations of several metabolites linked with peptidoglycan synthesis were grossly increased in Δ*metB* and Δ*metNIQ*Δ*metB* (Fig. 5*D*). Enterobacteriaceae peptidoglycan usually consists of alternating molecules of GlcNAc and *N*-acetylmuramic acid that are linked by a tetrapeptide of l-alanine, d-glutamate, diaminopimelic acid (DAP), and d-alanine. Because Met biosynthesis and *m*-DAP biosynthesis are linked through aspartate metabolism (43) and l-cystathionine can substitute for DAP (51), the perturbation of the intracellular metabolite pools related to Met biosynthesis might also impact peptidoglycan synthesis. Whereas LC-MS revealed significant changes in the levels of peptidoglycan intermediates, these changes were not reflected by increased sensitivity to agents (*e.g.* β-lactam antibiotics) that act through peptidoglycan synthesis or directly on peptidoglycan with the exception of growth in bile. Bile is known to remodel *S. enterica* peptidoglycan (52). However, it is recognized that Enterobacteriaceae with altered cell wall physiology are very robust. For example, *E. coli* with decreased *m*-DAP, which reduced the peptidoglycan density by 50%, did not show any detectable alteration in morphology or growth characteristics (53).

The data obtained from the LC-MS analysis with the complemented mutant Δ*metNIQ*Δ*metB* (Figs. 4 and 5) suggested that complementation by pACYC184*metB* did not fully restore the metabolite levels seen in the Δ*metNIQ* mutant. However, this is not unusual because, as related, genetic complementation using a plasmid will differ from the native level due to plasmid copy number and regulation, accounting for the partially complemented phenomenon that is observed.

This study supports earlier models of *S. enterica* metabolism that have suggested significant redundancy in key pathways linked with growth (13). The research reported here was a systematic analysis of Met metabolism in response to earlier investigations, which suggested that Met auxotrophy was attenuating for bacterial growth in animals and that *de novo* Met metabolism might therefore provide new antibiotic targets. The regulation of Met synthesis, a complex regulon comprising MetR and MetJ (16, 54), was not investigated because the aim of the study was to determine the essentiality of *de novo* Met synthesis in *in vivo* growth. We found that mutants unable to synthesize Met efficiently obtained the amino acid from their intracellular niche via their high-affinity Met transporter, suggesting that the SCV contains sufficient Met to sustain normal bacterial growth even in the absence of *de novo* synthesis. Severe reductions in virulence *in vivo* were only observed when both *de novo* Met biosynthesis and high-affinity Met transport were lost. The impact of mutations in the Met biosynthesis and transporter genes on other pathways was revealed, reaffirming the complexity of the bacterial metabolome and the interactions between metabolites that have yet to be mapped. Considerably more basic science on bacterial metabolism will be needed to identify novel, functionally nonredundant targets.

## Experimental procedures

### Bacterial strains and growth conditions

The bacterial strains and plasmids used in this study are listed in Table 2. All mutant strains were constructed on the *S.* Typhimurium SL1344 genetic background, and SL1344 was used as the WT strain in all experiments. The SL1344 strain has been described previously (36); its virulence is well defined and is resistant to streptomycin. Appropriate antibiotics, including streptomycin (50 μg/ml), chloramphenicol (30 μg/ml), kanamycin (50 μg/ml), and ampicillin (100 μg/ml), were added to growth medium as required. To obtain mid-exponential growth phase, *S*. Typhimurium and *E. coli* strains were grown in 10 ml of LB broth (BD Difco) overnight, and 100 μl of the overnight culture was subcultured into 10 ml of fresh LB broth and grown with shaking (180 rpm) at 37 °C for 3–4 h until the optical density reading at 600 nm reached 0.6–0.8. Growth phenotypes were characterized in LB broth or M9 minimal medium (2 mm MgSO_4_, 0.1 mm CaCl_2_, 0.4% glucose, 8.5 mm NaCl, 42 mm Na_2_HPO_4_, 22 mm KH_2_PO_4_, 18.6 mm NH_4_Cl, and 100 μm histidine). Met or vitamin B_12_ was supplemented at 100 μm. For assessing growth under anaerobic conditions, cultures were grown shaking in air-tight jars with AnaeroGen (ThermoFisher) for depletion of oxygen.

**Table 2:** Strains and plasmids used for this study Str, streptomycin; Ap, ampicillin; Chl, chloramphenicol; Km, kanamycin; Ara, arabinose; Flp, flippase; FRT, fippase recombinase target.

Strain or plasmid	Relevant phenotypes and genotypes	Source/Reference
**Strains**		
*S. enterica* Typhimurium SL1344	Wild-type strain *rpsL hisG46*; Str^R^	Ref. 36
*S. enterica* Typhimurium BRD666	Restriction negative modification positive (r^−^m^+^) SL1344; Str^R^	Ref. 68
*E. coli* DH5α	Cloning strain	Ref. 69

**Plasmids**		
pGEM-T Easy	High-copy number cloning vector for PCR products; Ap^R^	Promega
pACBSR	Medium-copy number, mutagenesis plasmid; p15A ori; Ara-inducible I-SceI and λ Red recombinase; Chl^R^	Ref. 55
pACYC184	Medium-copy number cloning vector, p15A ori; Tet^R^, Chl^R^	Ref. 70
pACYC184*metB*	*S*. Typhimurium SL1344 *metB* cloned into pACYC184; Chl^R^	This study
pCP20	FLP recombinase, temperature-sensitive replicon; Ap^R^, Chl^R^	Ref. 71
pKD4	FRT-flanked Km^R^ cassette; Ap^R^, Km^R^	Ref. 72

### Construction of strains and plasmids

Defined deletions of sequence encoding the *met* biosynthesis genes *metA*, *metB*, *metC*, *metE*, *metF*, and *metH* and Met high-affinity transporter *metNIQ* were generated in *S*. Typhimurium SL1344. The biosynthetic mutant and high-affinity transporter mutant were combined together to generate the double mutant Δ*metNIQ*Δ*metB* and the triple mutant Δ*metNIQ*Δ*metEH.* The double mutant Δ*metNIQ*Δ*metB* was complemented by introducing the *de novo* biosynthetic *metB* gene into Δ*metNIQ*Δ*metB* strain in *trans*. This strain is named Δ*metNIQ*Δ*metB* pACYC184*metB.* Gene deletions and concomitant insertions of an antibiotic resistance cassette were constructed using a Lambda Red-mediated “gene gorging” method (55). All constructs were verified by PCR and moved to an SL1344 or relevant background via P22 phage transduction (36, 56). Primers used to construct mutants are listed in Table 3. Genetic complementation of mutant Δ*metNIQ*Δ*metB* was achieved by cloning *metB* into pACYC184 via the BamHI and SalI sites and then introducing pACYC184*metB* into the Δ*metNIQ*Δ*metB* mutant. All constructs were verified by restriction analysis and DNA sequencing. Key mutants were also sequenced using Illumina whole-genome sequencing and analyzed using the pipeline at the Wellcome Sanger Institute (Hinxton, UK).

**Table 3:** Oligonucleotide primers used in this study for construction of mutants and complementation Endonuclease restriction sites are underlined; kanamycin resistance cassette-specific sequences are in boldace type. F, forward (5′) primer; R, reverse (3′) primer.

Mutants	Sequence (5′–3′)
Δ*metA-*ISceI*-*F	TAGGGATAACAGGGTAATCGCCAGTGTTAACGCATGTTC
Δ*metA-*ISceI-R	TAGGGATAACAGGGTAATCGGAATACCACGAATCTGCC
Δ*metA*-Kan-F	**CTAAGGAGGATATTCATATG**CGCAGCCACGGTAATTTACTG
Δ*metA*-Kan-R	GAAGCAGCTCCAGCCTACACAACCTGATAACCTCACGACATACG
Δ*metB-*ISceI-F	TAGGGATAACAGGGTAATCGCAGATCGGCATCATCC
Δ*metB*-ISceI-R	TAGGGATAACAGGGTAATCTTCATCAACCTGCGGCTG
Δ*metB*-Kan-F	**CTAAGGAGGATATTCATATG**CGGTATTGAAGATGGCGAAG
Δ*metB*-Kan-R	**GAAGCAGCTCCAGCCTACACA**CAGCCGTATTGTTCGTCATCG
Δ*metC*-ISceI-F	TAGGGATAACAGGGTAATCCTTCGTTATCTTCGCTGCC
Δ*metC-*ISceI-R	TAGGGATAACAGGGTAATCAGCAGAGTGCGGACAAACG
Δ*metC*-Kan-F	**CTAAGGAGGATATTCATATG**GCTGGTTCGGGTGCATATTG
Δ*metC*-Kan-R	**GAAGCAGCTCCAGCCTACACA**CACTATTCACTGAGCCAAGCG
Δ*metE-*ISceI-F	TAGGGATAACAGGGTAATCTACCTGCGGCCAGCTTG
Δ*metE*-ISceI-R	TAGGGATAACAGGGTAATCAATGCGGTCGCCACTCTG
Δ*metE*-Kan-F	**CTAAGGAGGATATTCATATG**GGCGTTAGCGAACATGGTC
Δ*metE*-Kan-R	**GAAGCAGCTCCAGCCTACACA**TCAACTCGCGACGCAGG
Δ*metF*-ISceI-F	TAGGGATAACAGGGTAATGCAGCCTGATGGAGCATGG
Δ*metF*-SceI-R	TAGGGATAACAGGGTAATGCCACGACCATCAATAGAACG
Δ*metF*-Kan-F	**CTAAGGAGGATATTCATATG**GCCGTGAAGGAGTGAAGGA
Δ*metF*-Kan-R	**GAAGCAGCTCCAGCCTACACA**CTTCCGCCAGGCTCTGATTC
Δ*metH*-ISceI-F	TAGGGATAACAGGGTAATCGGTGAGTCGTGGAATTAGGC
Δ*metH*-ISceI-R	TAGGGATAACAGGGTAATCGTCAGGGCGACAAGATCC
Δ*metH*-Kan-F	**CTAAGGAGGATATTCATATG**GAGGATGTTGAGCGGTGGC
Δ*metH*-Kan-R	**GAAGCAGCTCCAGCCTACACA**GCCGTCCAGCACCAGAATAC
Δ*metNIQ*-ISceI-F	TAGGGATAACAGGGTAATCACAGCTGTGCAGCAGG
Δ*metNIQ*-ISce-R	TAGGGATAACAGGGTAATACTGCCCTGCGGATGG
Δ*metNIQ*-Kan-F	**CTAAGGAGGATATTCATATG**TCCCCTGCTGGAACACTT
Δ*metNIQ*-Kan-R	**GAAGCAGCTCCAGCCTACACA**GTCTGATGAAGTGTACGAAGCC
*metB*-BamHI-F	TGGATCCGTCGCAGATGTGCGCTAATG
*metB*-SalI-R	TGTCGACCATAATGCCTGCGACACGC

### Infection of epithelial cells

Infection of HeLa cells (sourced from American Type Culture Collection (ATCC)) was conducted using established gentamicin protection assay protocols (57, 58). Briefly, HeLa cells were grown in DMEM supplemented with 10% fetal calf serum and 2 mm l-GlutaMAX (Life Technologies), in a humidified 37 °C, 5% CO_2_ incubator. One day before infection, HeLa cells were seeded in 24-well plates at 2 × 10^5^ cells/well. *S.* Typhimurium strains were grown to mid-exponential phase before and were frozen in LB with 10% glycerol at −80 °C. Bacteria were thawed immediately before infection, washed in antibiotic-free tissue culture medium, and diluted in DMEM with or without l-Met and then added to HeLa cell monolayers at a multiplicity of infection (MOI) of 5–10. The cfu in the inoculum was estimated by plating on LB agar plates. Infected HeLa cells were centrifuged at 600 × *g* for 5 min immediately after the addition of bacteria and then incubated for 1 h at 37 °C. After 1 h, the tissue culture medium was replaced with DMEM with or without Met and containing 100 μg/ml gentamicin to kill extracellular bacteria. The concentration of gentamicin was reduced to 10 μg/ml at 2 h post-infection and maintained for the remainder of the experiment. To enumerate intracellular bacteria, cells were washed twice with PBS and lysed with 1% Triton X-100 (Sigma) for 15 min to release the intracellular bacteria. The bacteria were enumerated by plating appropriate dilutions on LB agar plates.

### Ethics statement

All animal research conducted in this study was approved by the Animal Ethics Committee (Biochemistry and Molecular Biology, Dental Science, Medicine, Microbiology, and Immunology) at the University of Melbourne, under project number 1413141. All experiments were conducted in accordance with the Australian Code of Practice for the Care and Use of Animals for Scientific Purposes, 8th edition, 2013.

### Mouse infections

Age- and sex-matched C57BL/6 mice were used between 6 and 8 weeks of age to assess the virulence of mutant strains, using either the intravenous or oral route of infection. For intravenous infections, 200 cfu of each bacterial strain was prepared in 200 μl of PBS and injected into the lateral tail vein. For oral infections, mice were orally gavaged with 100 μl of 10% sodium bicarbonate immediately before oral gavage of 200 μl containing ∼5 × 10^7^ cfu of *S*. Typhimurium strains. To prepare the inoculum, all strains of *S*. Typhimurium were grown shaking at 180 rpm in M9 minimal medium supplemented with 100 μm Met, at 37 °C for 24 h, and stored in 10% glycerol at −80 °C until use. Immediately before infection, the stored aliquots were thawed and diluted in PBS to the required concentration. At designated time points post-infection, the spleen and liver were removed aseptically, homogenated using the Stomacher 80 Biomaster paddle blender (Seward), and serial dilutions were plated on LB agar plates with streptomycin to determine the bacterial load.

### Preparation of stock solutions and standards for LC-MS

Stock solutions of the related underivatized metabolites were prepared at 1.0 mg/ml in an appropriate solvent. Before using, the solutions were combined and diluted with water to give an appropriate standard metabolite mix solution. ^13^C^15^N-Aspartate (Cambridge Bioscience) was used as an internal standard at 1 μm in Milli-Q water. All stock solutions were stored at −20 °C.

### Sample harvest (metabolic arrest) for LC-MS

Each *S.* Typhimurium strain was grown at 37 °C to mid-exponential phase in 10 ml of LB, with shaking at 180 rpm. The culture was diluted into 30 ml of PBS and chilled in an ice/water slurry for 5 min and subsequently centrifuged (931 × *g*, 1 °C, 10 min). The pellet was then washed in 1 ml of PBS and centrifuged (17,295 × *g*). This washing step was repeated before removing the PBS and storing the cell pellet at −80 °C until metabolite extraction was performed.

### Extraction of metabolites for LC-MS

Cell pellets were resuspended with 400 μl of 75% ethanol solution containing 1:1000 of internal standard. Cell lysis was ensured by repeated cycles of freeze-thaw, for a total of 10 times, while cooling to −80 °C. Cellular debris was pelleted by centrifugation (17,295 × *g*, 5 min, 1 °C). The metabolic extract was transferred to a new tube and stored at −80 °C until analysis.

### Instrumentation

A SeQuant ZIC-pHILIC column (5 μm, 150 × 4.6 mm; Millipore) coupled to a 1260 series HPLC system (Agilent) was used to separate metabolites. The method used was described previously (59) with slight modifications; a flow rate of 0.3 ml/min with 20 mm ammonium carbonate in water and 100% acetonitrile was used as the mobile phase. A binary gradient was set up as follows: 0.5 min, 80% acetonitrile; 15.5 min, 50% acetonitrile; 17.5 min, 20% acetonitrile; 18.5 min, 5% acetonitrile; 21 min, 5% acetonitrile; 23 min, 80% acetonitrile; held at 80% acetonitrile until 29.5 min. Detection of metabolites was performed on an Agilent Q-TOF mass spectrometer 6545 operating in negative ESI mode. The scan range was 85–1200 *m*/*z* between 2 and 28.2 min at 0.8 spectra/s.

### Calibration and validation

LC-MS.d files were converted to .mzXML files using MS convert and analyzed using the LCMS R package (60, 61). Following alignment, groups were extracted with a mass window of 10 ppm, and statistical analysis was performed using MetaboAnalyst version 3.0 (62). The data set was uploaded, filtered using the interquartile range, and log-transformed, and a one-way ANOVA was performed with Tukey's honestly significant difference test (*p* < 0.01). Data were analyzed using MAVEN in parallel to validate the LC-MS results (63). Following alignment, metabolites were assigned using exact mass (<10 ppm) and retention time (compared with a standards library of 150 compounds run the same day). Scatter plots were generated for each pairwise comparison, and statistical significance was determined using a *p* value < 0.05 with Benjamini correction.

## Author contributions

A. U. H. and R. A. S. conceptualization; A. U. H., N. W., S. A. C., H. J. N., J. J. T., M. J. M., and R. A. S. formal analysis; A. U. H., N. W., S. A. C., D. M. H., J. J. W., T. A. S., M. R. D., and J. C. H. investigation; A. U. H., N. W., S. A. C., H. J. N., D. M. H., J. J. W., T. A. S., M. R. D., J. C. H., M. J. M., and R. A. S. methodology; A. U. H. and S. A. C. writing-original draft; N. W., H. J. N., M. J. M., and R. A. S. supervision; N. W. and J. J. T. visualization; N. W., H. J. N., T. L., M. J. M., and R. A. S. writing-review and editing; T. L., M. J. M., and R. A. S. funding acquisition.

## Supplementary Material

Supporting Information

## Footnotes

7 The abbreviations used are:

SAM: *S*-adenosylmethionine
NTS: nontyphoidal *Salmonella*
SCV: *Salmonella-*containing vacuole
LB: Luria broth
DMEM: Dulbecco's modified Eagle's medium
cfu: colony-forming unit(s)
DAP: diaminopimelic acid
MOI: multiplicity of infection
ANOVA: analysis of variance.
