# Supplementary material for: Methionine biosynthesis and transport are functionally redundant for the growth and virulence of Salmonella Typhimurium
Source: J Biol Chem. 2018 May 2;293(24):9506–19. doi: 10.1074/jbc.RA118.002592 (PMC6005444; doi:10.1074/jbc.RA118.002592)
Supplement: Supporting Information [file supp_293_24_9506__index.html]

Methionine biosynthesis and transport are functionally redundant for the growth and virulence of Salmonella Typhimurium — Methionine in Salmonella Typhimurium virulence — Supporting Information 

# Methionine biosynthesis and transport are functionally redundant for the growth and virulence of *Salmonella* Typhimurium

## Supporting Information

- JBC-2018-002592 Husna et al Supporting Information - This file contains supporting information, including one table and five figures
- JBC-2018-002592 Husna et al Supporting Information main text - This is the Word file for the Supporting Information, contains the table and figure legends only, no figures included
