# Supplementary material for: Methionine biosynthesis and transport are functionally redundant for the growth and virulence of Salmonella Typhimurium
Source: J Biol Chem. 2018 May 2;293(24):9506–19. doi: 10.1074/jbc.RA118.002592 (PMC6005444; doi:10.1074/jbc.RA118.002592)
Supplement: Supporting Information [file supp_RA118.002592_136246_1_supp_129713_p7x5yw.pdf]

SUPPLEMENTARY INFORMATION

Methionine biosynthesis and transport are functionally redundant for the growth and virulence of *Salmonella* Typhimurium

Asma Ul Husna[1], Nancy Wang[1*], Simon A. Cobbold[2], Hayley J. Newton[1], Dianna M. Hocking[1], Jonathan J. Wilksch[1], Timothy A. Scott[1,3], Mark R. Davies[1], Jay C. Hinton[4], Jai J. Tree[1,5], Trevor Lithgow[6], Malcolm J. McConville[2^], Richard A. Strugnell[1*]

Materials included:

Table S1: Survival analysis of *S.* Typhimurium wild-type and Δ*metB* mutant in different types of media supplemented with a variety of substances for different times

Supplementary Figure S1: Expression of genes in the *de novo* Met biosynthetic pathway of *S.* Typhimurium during stresses in relevant to *in vivo* infection

Supplementary Figure S2: The presence of Met in tissue culture DMEM media restores the growth of mutants in the *de novo* Met biosynthesis pathway in HeLa cells

Supplementary Figure S3: *De novo* Met biosynthetic mutants are not attenuated for oral infection in mice.

Supplementary Figure S4: *S.* Typhimurium mutants with combined deficiency for biosynthesis and high-affinity transport of Met are attenuated for oral infection in mice.

Supplementary Figure S5: *S.* Typhimurium Δ*metB* mutant shows slower growth in high concentration of bile salt.

Table S1. Survival analysis of *S.* Typhimurium wild-type and Δ*metB* mutant in different types of media supplemented with a variety of substances for different times.

**Different types of substances with different time-point**	**Difference in viable counts between wild-type and Δ*metB***
3.5% SDS, in LB, incubated for 2, 4, 6, 8 and 10 hours	No difference
0.5 mM EDTA, in LB, for 2, 4, 6, 8 and 10 hours	No difference
3.5% SDS and 0.5 mM EDTA, in LB, for 2, 4, 6, 8 and 10 hours	No difference
Different lysozyme concentration (0.5, 1, 2, 4, 8, 16 mg/ml) in LB, incubated for 10, 20, 30, 40, 60, 80 and 110 min after treatment with 0.5 mM EDTA	No difference
Different lysozyme concentration (0.5, 1, 2, 4, 8, 16 mg/ml) in LB, incubated for 10, 20, 30, 40, 60, 80 and 110 min after treatment with 1 mM EDTA	No difference
Distilled water for ten days	No difference
Fasted State Simulated Intestinal Fluid (FaSSIF) for seven days	No difference

Figure legends

Supplementary Figure S1. Expression of genes in the *de novo* Met biosynthetic pathway of *S.* Typhimurium during stresses in relevant to *in vivo* infection. Expression data were compiled from the SalComMac database (http://bioinf.gen.tcd.ie/cgi-bin/salcom.pl?db=salcom_mac_HL) as reported by Kröger *et al* (29) and Srikumar *et al.* (30), and provide detailed descriptions of media composition and stress. **A)** Absolute expression values for genes in the *de novo* Met biosynthetic pathway (transcripts per million). Growth conditions indicated below the heatmap are EEP (early exponential phase), MEP (mid exponential phase), LEP (late exponential phase), ESP (early stationary phase), LEP (late stationary phase), NonSPI2 (growth in PCN media (73) [pH 7.4 25mM Pi] to OD_{600} = 0.3), InSPI2 (growth in PCN medium [pH 5.8, 0.4 mM Pi] to OD_{600} = 0.3), InSPI2 low Mg^{2+} (growth in PCN medium [InSPI2] with 10 mM $MgSO_4$ to OD_{600} = 0.3), Intra-macrophages (infection of RAW264.7 macrophages for 8 hours, at a bacteria:macrophage ratio of 100:1). **B)** Relative expression (log_2 fold change) of genes in the *de novo* Met biosynthetic pathway. Gene expression is shown relative to the first column in each block of samples (designated "normalizer"). Stress conditions are as described for absolute expression (A) and described in detail in (29).

Supplementary Figure S2. The presence of Met in tissue culture DMEM media restores the growth of mutants in the *de novo* Met biosynthesis pathway in HeLa cells. HeLa cells were grown to a monolayer and infected with *S.* Typhimurium WT or mutant strains at a multiplicity of infection (MOI) of 5-10, in DMEM-complete media that contains 200 μM Met. The intracellular bacterial load at 2 hrs post-infection is expressed as "1" and used as the reference point to calculate fold-change of intracellular bacterial number at subsequent time points. Data are pooled from three independent experiments. Bars represent the mean cfu and error bars show the data range. Unpaired *t*-test was used to compare the intracellular load of WT and mutant strains at 10 hrs post-infection, and multiple comparisons were corrected using the Bonferroni-Dunn method; none of the comparisons yielded a *p*-value below 0.05.

Supplementary Figure S3. *De novo* Met biosynthetic mutants are not attenuated for oral infection in mice. C57BL/6 mice were oral gavaged with 10% sodium bicarbonate immediately before oral gavage with 5×10^7cfu of indicated strains of *S.* Typhimurium. The bacterial load in the A) liver and B) spleen were determined at day 6 post-infection. Symbols represent data from individual animals, and horizontal lines represent the geometric mean of each group. One-way ANOVA with Bonferroni post-tests was used for statistical analyses comparing each pair of data groups, and none of the comparisons yielded a *p*-value below 0.05.

Supplementary Figure S4. *S.* Typhimurium mutants with combined deficiency for biosynthesis and high-affinity transport of Met are attenuated for oral infection in mice. C57BL/6 mice were oral gavaged with 10% sodium bicarbonate immediately before oral gavage with 5×10^7cfu of indicated strains of *S.* Typhimurium. The bacterial load in the A) liver and B) spleen were determined at day 6 post-infection. Symbols represent data from individual animals, and horizontal lines represent the geometric mean of each group. Data are pooled from three independent experiments. One-way ANOVA with Bonferroni post-tests was used for statistical analyses comparing each pair of data groups, and none of the comparisons yielded a *p*-value below 0.05.

Supplementary Figure S5. *S.* Typhimurium Δ*metB* mutant shows slower growth in high concentration of bile salt. The growth of *S.* Typhimurium wild-type and Δ*metB* mutant was tested in MacConkey broth supplemented with 0.6% bile salt. Bars represent the mean optical density reading at 600 nm (OD_{600}) of three biological replicates for each strain, and error bars indicate the data range. Two-way ANOVA with Bonferroni's multiple comparison test was used for statistical analyses, ***, $p<0.001$; ****, $p< 0.0001$; ns, $p>0.05$.

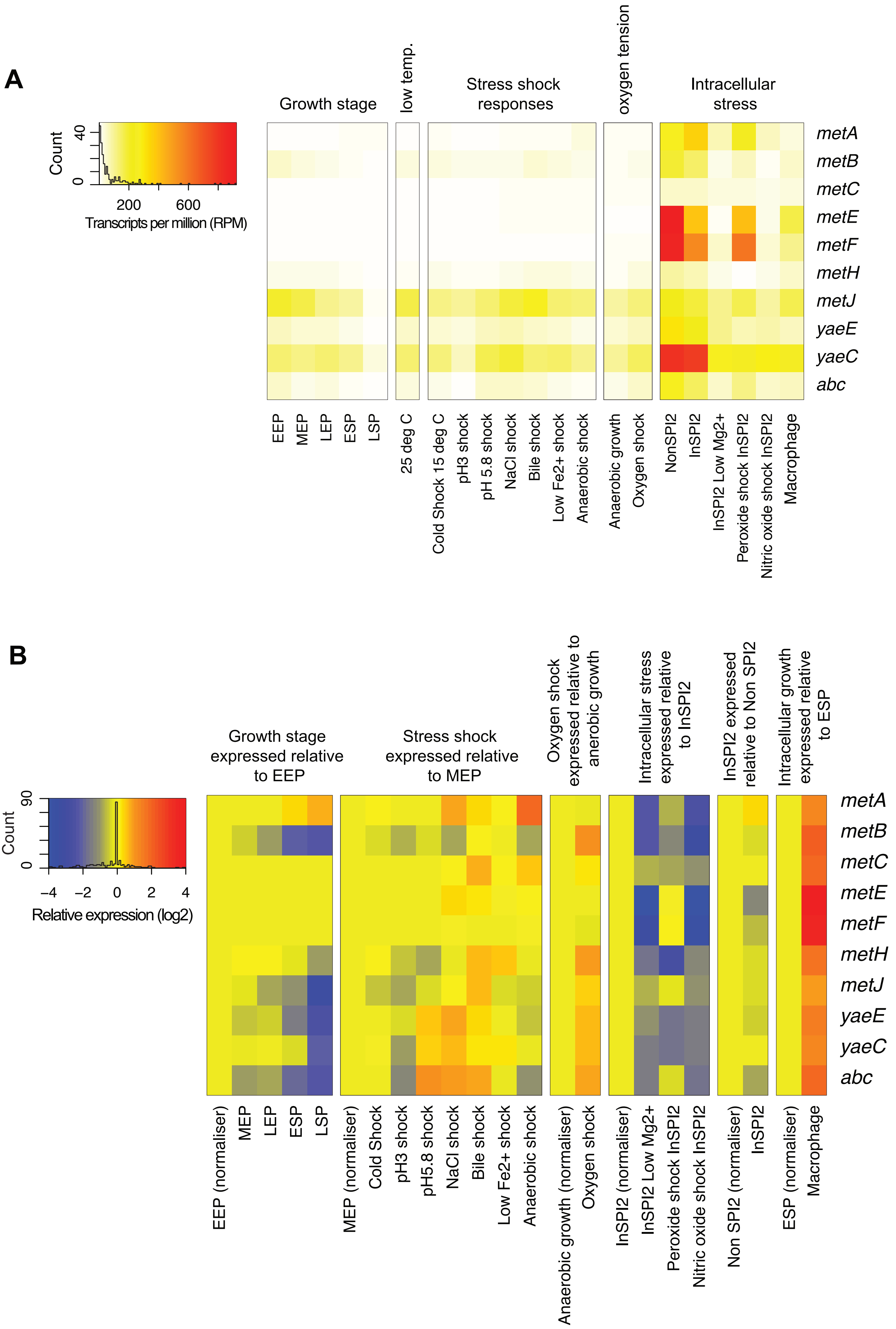
A
Count
0
40
200
600
Transcripts per million (RPM)
Growth stage
low temp.
Stress shock responses
oxygen tension
Intracellular stress
metA
metB
metC
metE
metF
metH
metJ
yaeE
yaeC
abc
EEP
MEP
LEP
ESP
LSP
25 deg C
Cold Shock 15 deg C
pH3 shock
pH 5.8 shock
NaCl shock
Bile shock
Low Fe2+ shock
Anaerobic shock
Anaerobic growth
Oxygen shock
NonSPI2
InSPI2
InSPI2 Low Mg2+
Peroxide shock InSPI2
Nitric oxide shock InSPI2
Macrophage
B
Count
0
90
−4
−2
0
2
4
Relative expression (log2)
Growth stage expressed relative to EEP
Stress shock expressed relative to MEP
Oxygen shock expressed relative to anerobic growth
Intracellular stress expressed relative to InSPI2
InSPI2 expressed relative to Non SPI2
Intracellular growth expressed relative to ESP
metA
metB
metC
metE
metF
metH
metJ
yaeE
yaeC
abc
EEP (normaliser)
MEP
LEP
ESP
LSP
MEP (normaliser)
Cold Shock
pH3 shock
pH5.8 shock
NaCl shock
Bile shock
Low Fe2+ shock
Anaerobic shock
Anaerobic growth (normaliser)
Oxygen shock
InSPI2 (normaliser)
InSPI2 Low Mg2+
Peroxide shock InSPI2
Nitric oxide shock InSPI2
Non SPI2 (normaliser)
InSPI2
ESP (normaliser)
Macrophage

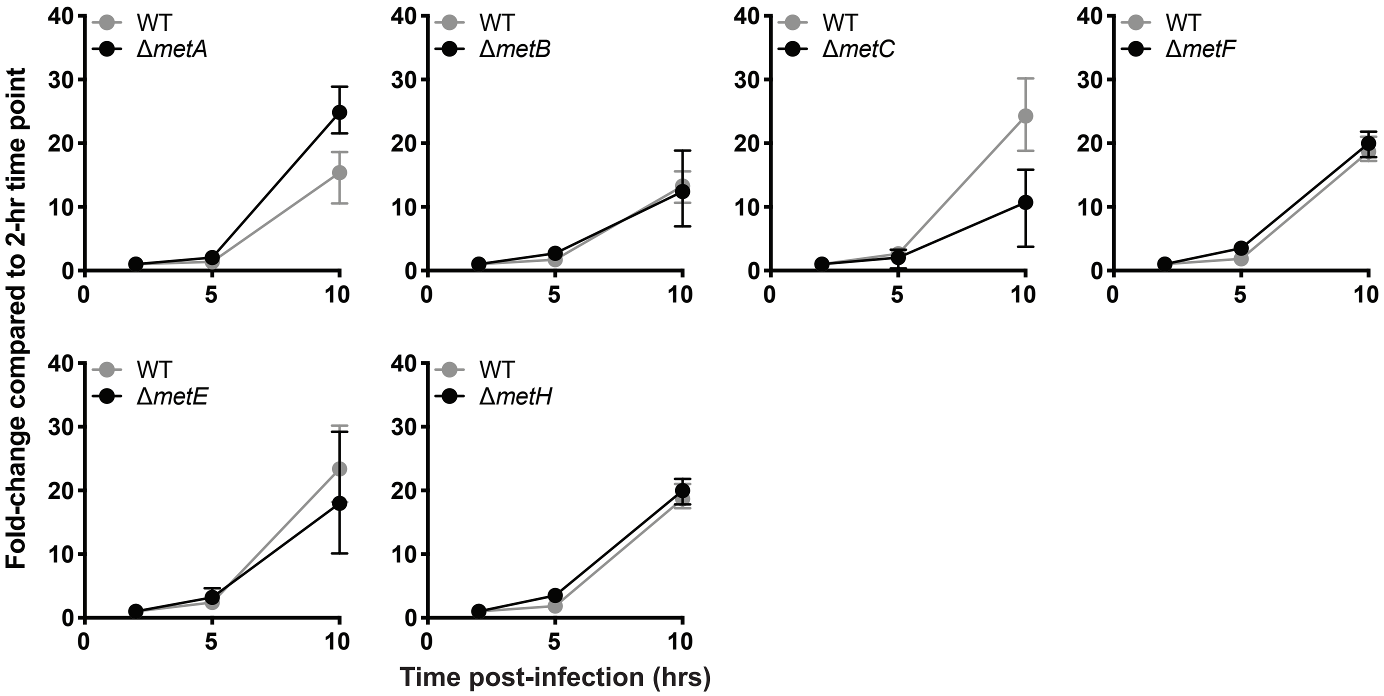
Fold-change compared to 2-hr time point
Time post-infection (hrs)
WT
ΔmetA
ΔmetB
ΔmetC
ΔmetF
ΔmetE
ΔmetH
0
5
10
20
30
40

Supp Fig. S2

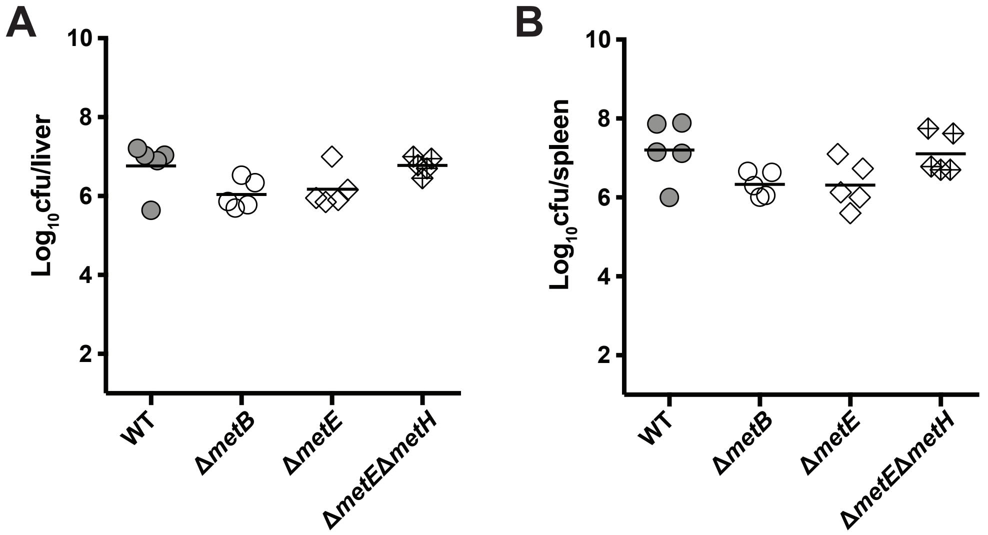
A
B
10
8
6
4
2
Log10cfu/liver
Log10cfu/spleen
WT
ΔmetB
ΔmetE
ΔmetEΔmetH

Supp Fig. S3

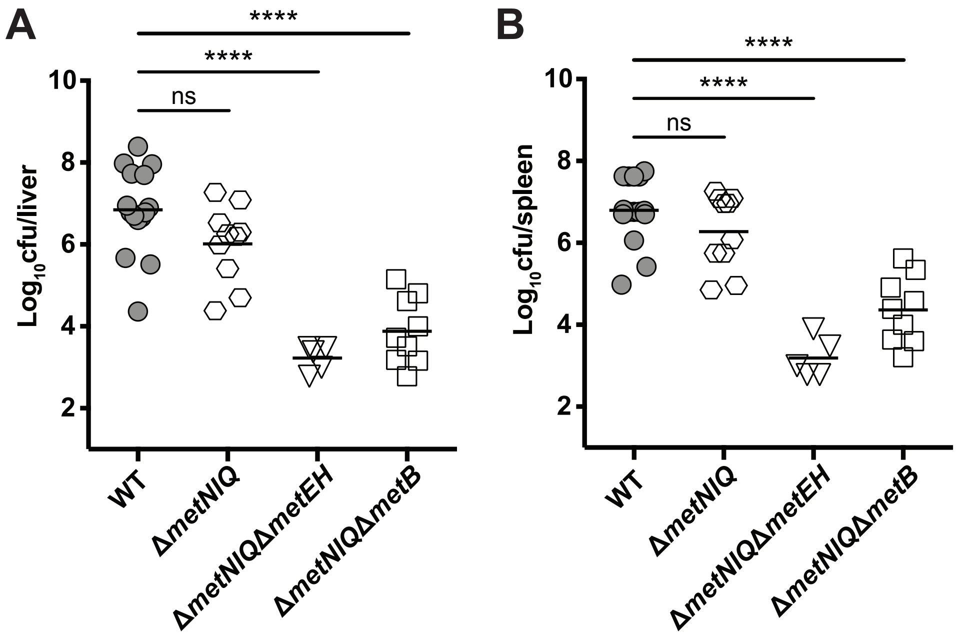
A
B
****
****
ns
10
8
6
4
2
Log10cfu/liver
Log10cfu/spleen
WT
ΔmetNIQ
ΔmetNIQΔmetEH
ΔmetNIQΔmetB

Supp Fig. S4

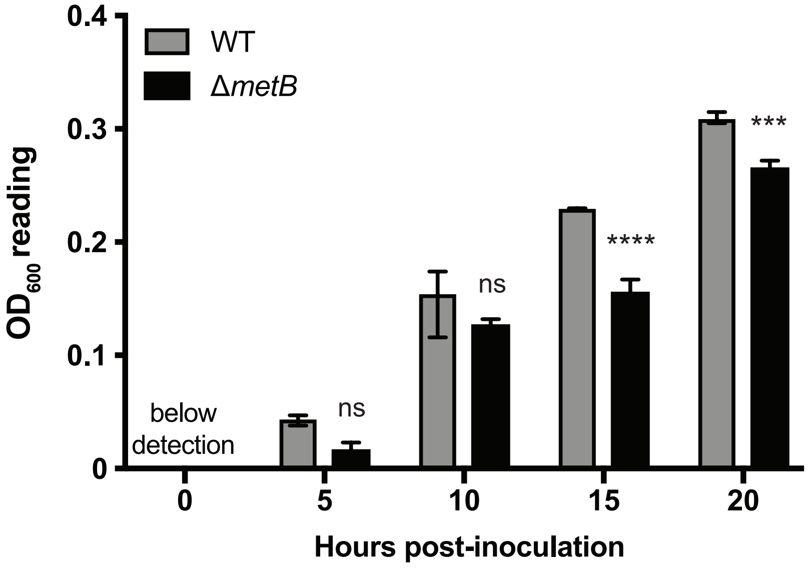
0.4
0.3
0.2
0.1
0
OD600 reading
WT
ΔmetB
below
detection
ns
ns
****
***
0
5
10
15
20
Hours post-inoculation

Supp Fig. S5
